# Author's Reply. RE: Routine clozapine assay monitoring to improve the management of treatment-resistant schizophrenia

**DOI:** 10.1192/bjb.2023.6

**Published:** 2023-04

**Authors:** David Kitchen

**Affiliations:** Senior Clinical Pharmacist, Mersey Care NHS Foundation Trust, UK. Email: david.kitchen2@nhs.net

I would very much like to thank Sumeet Gupta and Liyana Nur Mohamad for their supporting comments. Hopefully, there are many more like-minded clinicians who would also wish to see further developments in this area. The potential benefits to all involved are truly enormous.

Clozapine is a unique and usually efficacious treatment for a significant group of the mental health population with treatment-resistant schizophrenia (TRS). However, in my experience, it would seem that only a fraction of the people who fulfil the criteria for a diagnosis of TRS are actually considered for clozapine treatment. The reasoning for this undertreatment is multifactored, but the general theme of various safety concerns with regard to the longer-term management of clozapine is invariably foremost in clinicians minds.

The robust moves we have made to ensure that clozapine therapeutic drug monitoring is a significant facet of every patients care plan have allowed us to: (a) identify previously unknown clinical risk and manage it carefully; (b) build a data-set of results for each patient, which is a helpful tool for overall clinical assessment; (c) improve clinical outcomes and reduce mortality; (d) improve the confidence of clinicians, which has allowed them to be more agile with their prescribing of clozapine; and (e) support clinicians to feel encouraged to consider more patients for clozapine, which is reflected in our above-average recruitment of patients for treatment.

I agree that the current provision for assay testing across the UK could be improved. However, the necessary laboratory technology has become more available over time, and I would encourage clinicians to develop dialogue with both national and local pathology laboratories to explore the potential service development.

Clozapine has been the gold standard treatment for TRS for many years, and yet it is still mostly underutilised. We need to address this serious shortfall in service provision.

